# Water-Soluble and Cytocompatible Phospholipid Polymers for Molecular Complexation to Enhance Biomolecule Transportation to Cells In Vitro

**DOI:** 10.3390/polym12081762

**Published:** 2020-08-06

**Authors:** Kazuhiko Ishihara, Shohei Hachiya, Yuuki Inoue, Kyoko Fukazawa, Tomohiro Konno

**Affiliations:** 1 Department of Materials Engineering, The University of Tokyo, 7-3-1 Hongo, Bunkyo-ku, Tokyo 113-8656, Japan; shohei.hachiya@astellas.com (S.H.); yuuki.inoue@lgjlab.com (Y.I.); fukazawa@mpc.t.u-tokyo.ac.jp (K.F.); 2 Graduate School of Pharmaceutical Sciences, Tohoku University, 6-3 Aoba-Aramaki, Aoba-ku, Sendai, Miyagi 980-8578, Japan

**Keywords:** 2-methacryloyloxyethyl phosphorylcholine polymer, amphiphilic nature, cationic group, polymer aggregate, endocytosis

## Abstract

Water-soluble and cytocompatible polymers were investigated to enhance a transporting efficiency of biomolecules into cells in vitro. The polymers composed of a 2-methacryloyloxyethyl phosphorylcholine (MPC) unit, a hydrophobic monomer unit, and a cationic monomer unit bearing an amino group were synthesized for complexation with model biomolecules, siRNA. The cationic MPC polymer was shown to interact with both siRNA and the cell membrane and was successively transported siRNA into cells. When introducing 20–50 mol% hydrophobic units into the cationic MPC polymer, transport of siRNA into cells. The MPC units (10–20 mol%) in the cationic MPC polymer were able to impart cytocompatibility, while maintaining interaction with siRNA and the cell membrane. The level of gene suppression of the siRNA/MPC polymer complex was evaluated in vitro and it was as the same level as that of a conventional siRNA transfection reagent, whereas its cytotoxicity was significantly lower. We concluded that these cytocompatible MPC polymers may be promising complexation reagent for introducing biomolecules into cells, with the potential to contribute to future fields of biotechnology, such as in vitro evaluation of gene functionality, and the production of engineered cells with biological functions.

## 1. Introduction

In the molecular design of a safe and effective water-soluble polymers for promoting the introduction of a biomolecule into cells, the interactions between the polymer and the biomolecule and that between the polymer and the cell membrane are key factors [1,2,3,4]. Most biomolecules have a complicated higher order structure, and have various functional groups, which may include hydrophilic, hydrophobic, anionic, cationic, and hydrogen-bonding groups, in a single molecule. Therefore, when focusing on the intermolecular interactions between the biomolecules and polymers, it is necessary to introduce a monomer unit that is capable of interacting with the target biomolecule [5,6,7]. For example, many cationic both lipids and polymers have been examined as transporter molecules for specific biomolecules into cells in vitro [3,8,9,10]. The transporter molecules must show enhanced efficiency of bioactivity and low cytotoxic activity. When focusing on the interaction between cells and polymers, it is also necessary to consider the characteristics of the cell membrane, which is the first barrier to successful permeation. The cell membrane forms a phospholipid bilayer, with hydrophilic groups on the outside and hydrophobic groups on the inside. In addition, sialic acid and acidic phospholipids are present on its surface, causing a negative charge [11,12]. Therefore, an interaction between the cell and the polymer can be engineered by introducing a hydrophobic group or a cationic group into the polymer [13,14]. It is desirable that the transporter into which the biomolecule is introduced respects the need for the stability of the cell and of the biomolecule being transported. Polymers with a phospholipid polar group in the side chain, such as 2-methacryloyloxyethyl phosphorylcholine (MPC) polymer, are known to have excellent cytocompatibility [15,16,17,18] and are expected to suppress interactions with fragile biomolecules [19,20,21,22,23,24]. In our previous research, we found that a water-soluble, amphiphilic MPC polymer can permeate the living cell membrane by simple molecular diffusion [20,21], and that intracellular molecules are not leached out during this process [22]. This makes MPC polymers excellent candidates for the development of a transporter of biomolecules to introduce to cells. Based on this molecular design concept, several MPC polymers with a cationic group have been examined [23,24,25,26,27].

In this study, the siRNA molecule was used as a model biomolecule, which is a small, double-stranded RNA with 21−23 base pairs, and is a hydrophilic polyanion [28]. Therefore, we hypothesized that the introduction of a cationic group into water-soluble polymer, with the potential of interacting with the siRNA, may produce an effective intermolecular interaction [13,14]. We synthesized random-type, water-soluble MPC copolymers systemically by free radical polymerization, while changing the composition of monomer units, the molecular weight of the polymer, and the structure of the cationic group. Using these MPC polymers, the efficacy of siRNA transportation was evaluated in vitro, with respect to the chemical structure of each synthesized polymer.

## 2. Materials and Methods

### 2.1. Materials

MPC was purchased from NOF Co., Ltd., Tokyo, Japan, synthesized by a previously reported procedure [15,17]. The following monomers and reagents were used, after further purification: *n*-butyl methacrylate (BMA, Kanto Chemical Co. Inc., Tokyo, Japan), 2-aminoethyl methacrylate hydrochloride (AEMA, Polysciences Inc., Warrington, PA, USA), 2-(trimethylammonium)ethyl methacrylate (TMAEMA, Tokyo Chemical Industry Co. Ltd., Tokyo, Japan) fluorescein *o*-methacrylate (FLMA, Sigma-Aldrich, Co., St. Louis, MO, USA). The following were used without further purification: 2,2′-azobisisobutyronitrile (AIBN, Kanto Chemical Co., Inc., Tokyo, Japan) and tert-butyl peroxyneodecanoate (PB-ND) (70% solution in hydrocarbon liquid, NOF Co., Ltd., Tokyo, Japan), Cy3-labeled Universal Negative Control siRNA (Cy3-siRNA) (Nippon Gene Co., Ltd., Tokyo, Japan), Control siRNA duplex, Firefly Luciferase GL3 (GL3-siRNA) (Nippon Gene Co., Ltd.), 5× siRNA Buffer (Thermo Fisher Scientific Inc., Waltham, MA, USA), d-Luciferin Potassium Salt (FUJI FILM Wako Pure Chemical Industries, Osaka, Japan), and Lipofectamine RNAiMAX (Invitrogen, Carlsbad, CA, USA) [29]. Other solvents and reagents were used without further purification.

Cells for transportation (HeLa-Luc) were human cell lines, derived from cervical cancer cells (HeLa), that constitutively express the luciferase gene [30]. These were provided by Kataoka Laboratory, Department of Materials Engineering, University of Tokyo.

### 2.2. Polymerization Procedure

Polymers were synthesized by a conventional radical polymerization process [15,31,32]. For example, PMB37 was synthesized by copolymerization of MPC and BMA using PB-ND as a polymerization initiator and ethanol (EtOH) as a solvent. A polymerization solution was prepared so that the monomer concentration was 1.6 mol/L, the in-feed monomer ratio of MPC/BMA was 0.30/0.70, by mole fraction, and the initiator concentration was 55 mmol/L. The ethanol was bubbled with argon gas for 10 min prior to use. The polymerization solution was placed in a three-necked flask and polymerized while refluxing and bubbling argon gas in an oil bath at 60 °C. Polymerization was carried out at 60 °C for 3.0 h, followed by 70 °C for 1.0 h. After diluting the polymerization solution with ethanol, unreacted monomer and the polymerization initiator were removed by reprecipitation with a mixed solvent composed of diethyl ether/chloroform in a ratio of 90/10 by volume. After drying at reduced pressure for 1 day to remove the solvent, the purified product was recovered and dissolved in pure water. Dialysis was performed for 3 days in pure water using a dialysis membrane with a molecular weight cut-off of 3.5 kDa. After freeze-drying to remove water, the residue was dissolved again in pure water and dialyzed again, in pure water, for 3 more days with a dialysis membrane of the same specification as before, to remove residual monomers. An aqueous solution was freeze-dried two days to obtain solid PMB37. In the cases of other polymer syntheses, AIBN was used as a polymerization initiator instead of PB-ND and methanol/water mixture (MeOH/H_2_O) was used as a solvent. In order to determine the composition of the polymers obtained, ^1^H-NMR spectroscopic measurement was performed using a JNM-GX 270 (JEOL, Tokyo, Japan). As solvents, CD_3_OD (methanol-d4) was used for PMT37, PMT154, and C_2_D_5_OD (ethanol-d6) for PMB37. For other polymers, f-PMB37, and PMT127, a D_2_O (deuterium oxide)/C_2_D_5_OD mixture (20/80) was used. Each sample was prepared so that the concentration of the solvent was 0.65 mL per 10 mg of the polymer. Representative ^1^H-NMR spectra are shown in Figure S1.

A fluorescent monomer unit was introduced into PMB37, in order to synthesize f-PMB37 [20]. A polymerization solution was prepared so that the monomer concentration was 1.6 mol/L, the charging ratio of MPC/BMA/FLMA was 0.30/0.70/0.001 by mole fraction, and the initiator concentration was 55 mmol/L. After bubbling with argon gas for 10 min, the tube was sealed and polymerized in an oil bath of 60 °C for 18 h. After diluting the polymerization solution with ethanol, the unreacted monomer and the polymerization initiator were removed by reprecipitation with a mixed solvent of diethyl ether/chloroform at 90/10 by volume. After drying under reduced pressure for 1 day to remove the solvent, the purified product was dialyzed in the same manner as described above, to remove unreacted monomers. An aqueous solution was freeze dried to obtain solid f-PMB37.

**Figure 1:**
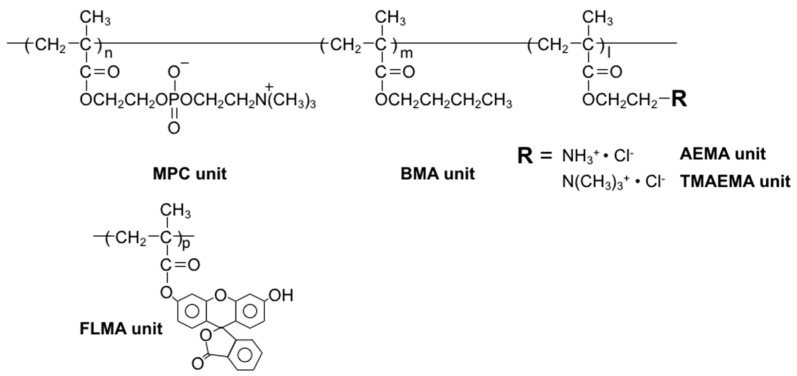
Chemical structure of 2-methacryloyloxyethyl phosphorylcholine (MPC) polymers used in this study.

Other polymers were also synthesized by the same procedure, while changing the monomer species and the compositions. The polymer structures are shown in Figure 1. Synthetic conditions and results for all polymers are summarized in Table 1.

### 2.3. Characterization of Polymers

The molecular weights of the polymers obtained were measured using gel permeation chromatography (GPC, JASCO, Tokyo, Japan) with a mixed solvent of methanol/water (70/30 by volume) containing 10 mM LiBr, using an SB-804 HQ (Shodex, Tokyo, Japan) column for PMB37 and f-PMB37, and a G6000PWXL-CP (Tosoh, Tokyo, Japan) column, for other polymers. Weight-averaged molecular weight (M_w_) and number-averaged molecular weight (M_n_) was determined using poly(ethylene glycol) (PEG, Tosoh, Tokyo, Japan) as a standard sample.

The fluorescence spectra of the polymers obtained were visualized using a fluorescence spectrophotometer (FP-8500, JASCO, Tokyo, Japan). The MPC polymer with the fluorescent monomer unit was dissolved in pure water at a concentration of 1 mg/mL. The fluorescence spectrum was measured in the range of 500–600 nm for the aqueous polymer solution. The excitation wavelength was 490 nm.

### 2.4. Surface Tension Measurement

The surface tension of the polymer solution was measured by the Wilhelmy method [32]. When a platinum plate (24 × 10 × 0.15 mm^3^) was immersed in the polymer solution at 25 °C, acting force was measured with a dynamic contact angle/surface tension measuring device (DCA-100, ORIENTEC, Co. Tokyo, Japan). Measurements were repeated at least five times, and the mean and standard deviations were determined.

### 2.5. Fluorescence Spectrum Measurement

The maximum fluorescence wavelength of sodium 8-anilinonaphthalene sulfonate (ANS) in aqueous polymer solution was measured using a fluorescence spectrophotometer (FP-8500, JASCO, Tokyo, Japan) at 25 °C [32]. The concentration of ANS was adjusted to 1.0 × 10^−5^ mol/L. The excitation wavelength was 350 nm and measurements were carried out at wavelengths in the range of 420–650 nm.

### 2.6. Cell Culture Procedure

The growth medium for cells was 10% fetal bovine serum (FBS) in Dulbecco’s modified Eagle’s medium (DMEM; Thermo Fisher Scientific Inc., Waltham, MA, USA). HeLa-Luc cells were seeded in a tissue culture polystyrene dish (TCPS) and cultured in growth medium, in a 5% CO_2_ atmosphere at 37 °C. When the cell density reached 80% of confluency, the growth medium was removed, cells were rinsed twice with phosphate-buffered saline (PBS), and then the cells were detached and collected using a cell detachment agent (TrypLE Express, Thermo Fisher Scientific Inc., Waltham, MA, USA). The cells were resuspended in growth medium, at an arbitrary concentration, and seeded on a TCPS.

### 2.7. Evaluation of Interaction of MPC Polymer with Cells

Fluorescence-labeled MPC polymers were used to evaluate the interaction between cells and polymer. HeLa-Luc cells were seeded on a glass dish at a density of 5.0 × 10^3^ cells/cm^2^. Pre-culturing of cells was carried out for 24 h at 37 °C, in a 5% CO_2_ atmosphere. After removing the culture medium and rinsing thrice with phosphate-buffered saline (PBS, pH 7.4, ionic strength 150 mM), the medium was exchanged with Opti-MEM in which MPC polymer was dissolved. After incubation for 20 min at 37 °C, in a 5% CO_2_ atmosphere, the glass dish was rinsed three times with PBS, then the microscopic observation of fluorescence was carried out, inside and around the cells in PBS, using a confocal laser microscope (Carl Zeiss, Oberkochen, Germany).

Cytotoxicity of polymers was evaluated by ATP-based luminescence assay as follows [33]. HeLa-Luc cells were seeded on 35 mm diameter TCPS at a density of 1 × 10^5^ cells/cm^2^. The cells were pre-cultured for 24 h in an atmosphere of 5% CO_2_ at 37 °C. After removing the growth medium and three times rinsing with PBS, the medium was exchanged with Opti-MEM. Moreover, d-luciferin was added as a luminescent substrate to a final concentration of 200 µg/mL. Then, the polymer solution was added to the medium. The concentration of polymers was adjusted to 1.0 mg/mL. Total volume of the culture medium was adjusted to 1.0 mL in each well. The emission intensity in the TCPS was measured for 48 h using a real-time luminescence measuring device (Kronos-dio AB-2550, ATTO Co., Ltd., Tokyo, Japan). Instead of polymer solution, commercially available reagent, Lipofectamine RNAiMAX was used. Concentration of contents in Lipofectamine RNAiMAX reagent has not been clarified. This is presumably because this reagent is mainly composed of cationic liposomes and is a molecular assembly. Therefore, a sample was prepared according to the manufacturer’s protocol used for gene transfer [29,34]. That is, 7.5 µL of the reagent was diluted by 250 µL of Opti-MEM and the solution was used for examination of cytotoxicity. Measurements were repeated at least three times.

### 2.8. Preparation of siRNA/MPC Polymer Complex

The formation of a complex between the MPC polymers PMB37, PMA37, and PMBA154M, and siRNA was evaluated by dynamic light scattering. The measurement was performed in two states, with and without addition of siRNA. The siRNA concentration was 600 nmol/L, the polymer concentration was 6.0 mg/mL for PMB37. In the cases of PMA37 and PMBA154M, the polymer concentration was adjusted by setting the ratio of the positive charge of each polymer against the negative charge of siRNA at 5/1. The mixtures of siRNA and polymer were prepared and incubated at room temperature for 15 min. As a control, the siRNA duplex, GL3-siRNA, was used. In addition, a polymer solution containing no siRNA was prepared at the same polymer concentration. Then, using a Zetasizer Nano (Malvern Instruments Co., Ltd., Worcestershire, UK), the average size of the polymer aggregate and the complex in each solution was measured. Also, ζ-potential of the complex in buffer solution (pH 7.4 phosphate buffer with 10 mM ionic strength) was measured. Measurements were repeated at least three times.

### 2.9. Introduction of siRNA/MPC Polymer Complex into Cells

The siRNAs used were Cy3-siRNA and GL3-siRNA. These were dissolved in RNase-free buffer solution prepared using 5× siRNA buffer (Thermo Fisher Scientific Inc., Waltham, MA, USA) and UltraPure DNase/RNase-Free distilled water (Invitrogen, Carlsbad, CA, USA) Then, 20 µmol/L siRNA solutions were prepared. Depending on the experiment, the siRNA solution was diluted with a serum-reduced medium Opti-MEM (Invitrogen) to the desired concentration, before use.

Polymer solutions were prepared by dissolving the polymer in Opti-MEM. In the case of PMBA154M, which would not completely dissolve in Opti-MEM, it was dissolved in ultrapure water. Regarding the cationic polymer, the concentration of polymer was determined according to the charge ratio with the siRNA. The charge ratio was fixed at 5/1, and concentration of the polymer was 1.2 × 10^5^ times higher than the concentration of siRNA. The concentration of other polymers was set to 10 mg/mL. Depending on the experiment, the polymer solution was diluted with Opti-MEM to the desired concentration, before use. Lipofectamine RNAiMAX was used as a positive control, in accordance with the manufacturer’s protocol [29,34]. The siRNA solution and the polymer solution, after adjustment for concentration, were mixed in a sterile tube. The mixed solution was incubated at room temperature for 20 min and then added to the culture dish to bring it into contact with the cells.

HeLa-Luc cells were seeded on a glass dish at a density of 5 × 10^4^ cells/cm^2^. The cells were pre-cultured for 24 h in an atmosphere of 5% CO_2_ at 37 °C. After removing the growth medium and rinsing with PBS three times, the medium was exchanged with Opti-MEM. At this time, the serum-free state was set. Then, the Cy3-labeled siRNA/MPC polymer complex solution was added to the medium and incubated for 20 min at 37 °C. The final concentration of siRNA was 50 nmol/L. The final concentration of the polymer was such that the ratio of positive charge on the polymer to the negative charge in the siRNA was 5/1. In the case of PMB37, the concentration was adjusted to 1.0 mg/mL. At a controlled time after the addition of the mixed solution, the fluorescence inside and around the cells was observed with a confocal laser scanning microscope.

In order to evaluate the transportation pathway of siRNA/MPC polymer complex, the uptake behavior of fluorescently labeled siRNA was observed at 4 °C [20]. HeLa-Luc cells were seeded on a glass dish at a density of 5 × 10^4^ cells/cm^2^, and pre-cultured for 24 h in an atmosphere of 5% CO_2_. After removing the growth medium and rinsing with PBS three times, the medium was exchanged with Opti-MEM, and incubated at 4 °C for 30 min. The mixed solution of polymer/siRNA was incubated at room temperature for 20 min, then at 4 °C for 5 min. The final concentration of siRNA was 50 nmol/L. The final concentration of the polymer was such that the ratio of the positive charge on the polymer to the negative charge on the siRNA was 5/1. The siRNA/MPC polymer complex solution was added to the medium and, after a controlled period of time, fluorescence inside and around the cells was observed using a confocal laser scanning microscope.

### 2.10. Evaluation of Functionality of siRNA/MPC Polymer Complex

HeLa-Luc cells were seeded on 35 mm diameter TCPS at a density of 1 × 10^5^ cells/cm^2^. The cells were pre-cultured for 24 h in an atmosphere of 5% CO_2_ at 37 °C. After removing the growth medium and rinsing with PBS three times, the medium was exchanged with Opti-MEM. At this time, the serum-free state was set. Also, d-luciferin was added as a luminescent substrate so that the final concentration was 200 µg/mL. Then, the GL-3-siRNA/polymer complex solution was added to the medium. The final concentration of siRNA was 50 nmol/L. The final concentration of the polymer was such that the ratio of the positive charge on the polymer to the negative charge on the siRNA was 5/1. Then, the emission intensity in the TCPS was measured for 48 h using a real-time luminescence measuring device.

In order to evaluate the siRNA transduction rate for each MPC polymer, luminescence was measured after varying the contact time between the GL-3-siRNA/MPC polymer complex solution and the cells. As a control, Lipofectamine RNAiMAX was used. HeLa-Luc cells were seeded and pre-cultured under the same conditions. After replacing the growth medium with Opti-MEM, the GL-3-siRNA/MPC polymer complex solution was added to the medium. The final concentrations of siRNA and polymer were set under the same conditions. After addition of the GL-3-siRNA/MPC polymer complex solution, the medium in the dish was removed at 1, 3, or 6 h to remove the polymer and siRNA. After removing the medium after 1 or 3 h, it was replaced with Opti-MEM, after rinsing three times with PBS. After 6 h, the medium was removed from all dishes, the dish was three times rinsed with PBS, and then the medium was replaced with DMEM containing 10% FBS and d-luciferin at 200 µg/mL. Then, using a real-time luminescence measuring device, the luminescence intensity in the dish was measured for 48 h.

### 2.11. Statistical Analysis

Data were presented as the mean ± SD of more than three independent experiments, the experiment groups were compared by one-factor analysis of variance (ANOVA) and followed by Student *t*-test. The significance of differences was evaluated for *p* < 0.01. All statistical analyses were performed using Microsoft Excel^®^ 2011 (Microsoft Corp., Redmond, WA, USA).

## 3. Results and Discussion

### 3.1. Characteristics of the MPC Polymers in Aqueous Medium

In this study, we obtained random-type copolymers. Every polymer synthesized were water-soluble and clear aqueous solutions were prepared. The solubilized morphology of polymers in aqueous solution is important when considering the interactions of polymer molecules with cells. In the case of amphiphilic block-type copolymers, they form micelle-like structures in an aqueous medium, through interaction between hydrophobic units [35,36,37]. There are many reports regarding polymeric micelles and their activities as drug transporters in biological systems [38,39,40]. However, in the case of amphiphilic random copolymers, although polymer aggregates can be formed in aqueous media, their structure is unstable and is dependent on the concentration of polymer [32]. We evaluated the process of association of the polymers used in this study, with regard to their chemical structure.

**Figure 2:**
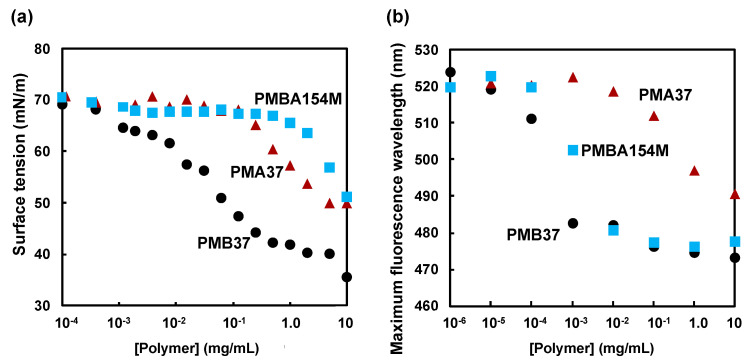
Polymer concentration dependence of (**a**) surface tension of polymer aqueous solution and (**b**) maximum fluorescence wavelength of polymer aqueous solution using sodium 8-anilinonaphthalene sulfonate (ANS) as fluorescence probe. Circle; PMB37, triangle; PMA37, and square; PMBA154M.

Figure 2a shows the dependence of surface tension of the polymer on its concentration in aqueous solution. For all three MPC polymers, the surface tension decreased when the polymer concentration exceeded a certain level. However, the point where the surface tension started to decrease varied between polymers. In the case of PMB37, this happened at a concentration of about 10^−2^ mg/mL, and became constant from about 1.0 mg/mL. This suggests that PMB37 formed an aggregate in which the hydrophilic phosphorylcholine group was oriented to the outside and the hydrophobic units were located inside. However, in PMA37 and PMBA154M, the decrease in surface tension occurred at a concentration of 10^−1^ mg/mL. The surface tension at 10 mg/mL was about 35 mN/m for PMB37, compared to 50 mN/m for PMA37 and PMBA154M. From these findings, we suggest that PMA37 and PMBA154M have less ability to lower surface tension, compared to PMB37. PMB37 was a copolymer of MPC and BMA, and PMBA154M was a copolymer of MPC, BMA and AEMA. Both MPC polymers are amphiphilic nature, with a hydrophilic unit and a hydrophobic unit in the molecule, and are considered to act as a surfactant in water [20,32]. PMBA154M has a M_w_ of approximately 3 × 10^5^, while that of PMB37 is about 1 × 10^4^. Since PMB37 has a relatively small molecular weight compared to PMBA154M, its ability to lower surface tension is greater. It has been reported that the PMB37 forms a thick hydration layer with high mobility [41]. Since PMB30 had a higher proportion of MPC units, compared to PMBA154M which has more AEMA units and fewer MPC units, it showed an affinity for water molecules, due to the hydration of the phosphorylcholine group. In summary, it was found that the MPC polymers exhibit different surfactant abilities, dependent on differences in their monomer units and molecular weight.

Figure 2b shows the concentration dependence of the maximum fluorescence wavelength of ANS in the polymer solutions. The wavelength of fluorescence reflects the polarity of the medium surrounding the ANS molecules [42]. We found that the wavelength of maximum fluorescence decreased when the polymer concentration exceeded a certain level, for the three types of MPC polymers used this study. In particular, PMB37 and PMBA154M showed a remarkable decrease in the wavelength of fluorescence, becoming almost constant at concentrations ≥ 10^−1^ mg/mL. From evidence of the fluorescence wavelength, there were some domains with polarity like ethanol [32]. PMB37 and PMBA154 are amphiphilic MPC polymers with hydrophobic groups. When this was taken together with the findings in Figure 2a, the following phenomena became clear. In these MPC polymer aqueous solutions, as the concentration of the solution increases, aggregates are formed in which the hydrophilic groups are oriented outside and the hydrophobic groups inside. It is also considered that ANS responds to the low-polarity environment inside the aggregate, causing a decrease in wavelength of the fluorescence peak. From these results, we concluded that PMB37 and PMBA154M start to aggregate at a concentration of 10^−3^ mg/mL or higher, and aggregation is complete at concentrations above 10^−1^ mg/mL.

### 3.2. Internalization of the MPC Polymers to Cells

**Figure 3:**
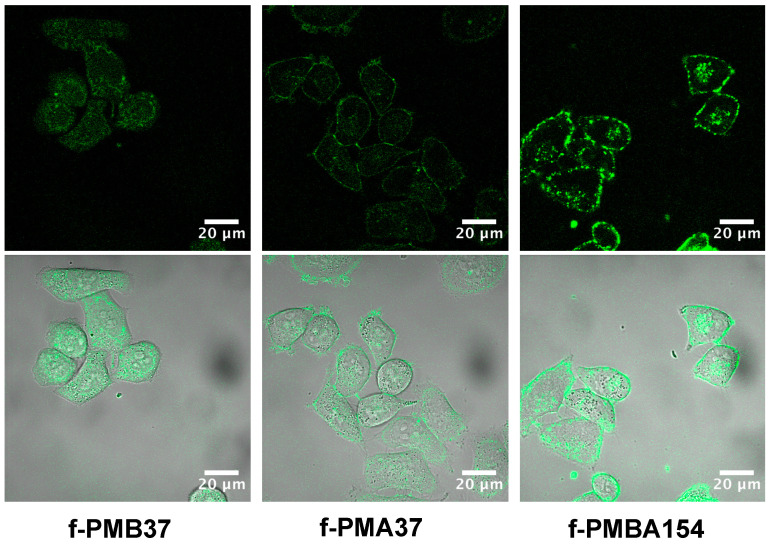
Laser confocal microscopic images of HeLa-Luc cells internalized, with various MPC polymers labeled with fluorescence monomer unit. Cells incubated in the medium containing the MPC polymer at 37 °C for 20 min.

Figure 3 shows the results of observation of the interaction between the fluorescent labeled MPC polymers and cells, using a confocal microscope. In all of three types of MPC polymer (f-PMB37, f-PMA37, and f-PMBA154), fluorescence derived from the MPC polymer was observed inside the cells. From this observation, it is considered that these MPC polymers interacted with the cell membrane and were taken up by the cells. In addition, a difference was observed in the fluorescence distribution, depending on the type of MPC polymer. In the case of f-PMB37, having a hydrophobic group, fluorescence was observed mainly inside the cells. From this finding, it is concluded that f-PMB37 was taken up into the cell by interacting with the cell via the hydrophobic group. In contrast, as f-PMA37 and f-PMBA154 have a cationic group, fluorescence was observed not only inside the cell but also in the vicinity of the surrounding cell membrane. These cationic MPC polymers adsorbed to the cell membrane by interaction with its negative charge. In addition, for these cationic MPC polymers, differences were observed in the distribution around the cell membrane and inside the cells. In the case of f-PMBA154, having a hydrophobic group, fluorescence with almost the same intensity was observed around the cell membrane and inside the cell. This polymer was easily taken into the cell through the lipid bilayer, due to the effect of its hydrophobic unit. However, f-PMA37 and f-PMBA154 were adsorbed on the negatively charged cell membrane, via the cationic unit of the polymer. It is considered that the hydrophobic BMA unit imparts affinity to the hydrophobic region of the cell membrane, while the cationic AEMA unit interacted with the negative charge on the membrane surface, in interactions between MPC polymer and cells.

**Figure 4:**
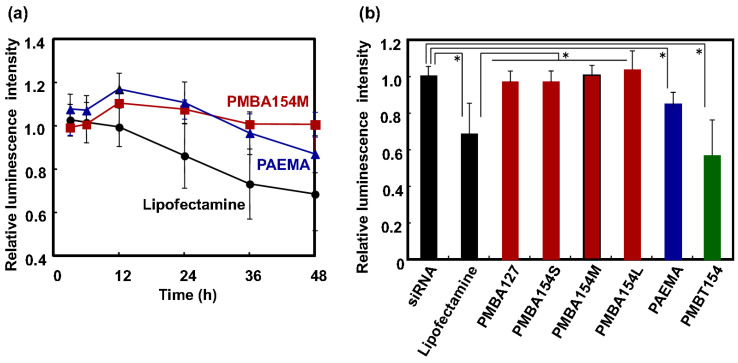
(**a**) Time course of relative luminescence intensity of HeLa-Luc cells on addition of lipofectamine and polymers. (**b**) Relative luminescence intensity after 48h-culturing with polymers (*n* = 3, SD). * represents *p* < 0.01.

We also evaluated the cytotoxicity of the polymers by an ATP-based luminescence assay [33]. Figure 4a shows a graph of the change in luminescence intensity over time when only the polymer was added. When the cell function is impaired, the amount of ATP produced by the cells and the amount of luciferase expressed in the cells are decreased, and the luciferase luminescence reaction is suppressed. Here, the luminescence intensity is a used as an indicator of cytotoxicity. When a homopolymer of AEMA (PAEMA) and a commercially available siRNA introduction reagent, Lipofectamine RNAiMAX, were added, the luminescence intensity decreased with time. However, when only PMBA154M was added, the rate of change in emission intensity was comparatively small. Cationic substances are known to damage to cells and to cause cytotoxicity [43,44]. Since PAEMA and Lipofectamine RNAiMAX are cationic polymers and lipids, we considered that cytotoxicity would occur. Although, the PMBA154M is a cationic polymer, the cytotoxicity has an MPC unit in the molecule. We suggest that the rate of change in luminescence intensity was small because its cytotoxicity, as a transporter, was suppressed by the effect of the MPC unit having a membrane-like structure. In the case of other MPC polymers, the relative emission intensity of cells, after 48 h contact with the polymers, is indicated in Figure 4b. Compared to PMBA154 series (with AEMA units), PMBT154 (with TMAEMA units) showed a larger decrease in relative intensity of luminescence. Xu et al. reported that a gene transporter composed of poly(N,N-dimethylaminoethyl methacrylate), which possesses a tertiary amino group in the side chain, has increased cytotoxicity when a part of the tertiary amino group is changed to a quaternary ammonium group [45]. The TMAEMA unit, a cationic quaternary ammonium group, has a positive charge regardless of the pH of the medium. In our previous report, we observed that the cytotoxicity of the substrates covered with the MPC polymers containing TMAEMA units depends on the TMAEMA unit composition when the TMAEMA units become over 0.6 [46]. That is, the cytotoxicity of the TMAEMA unit is confirmed. This suggests that the interaction between PMBT154 and cells was too strong for gentle transportation and, therefore, its cytotoxicity was higher than that of PMBA154. From this result, it is suggested that PMBA154 is a polymer transporter that has no adverse effect on cells and exhibits cell compatibility.

### 3.3. Complex Formation of the MPC Polymer with siRNA

Table 2 shows the measurement of volume-averaged aggregate size of the MPC polymers and Cy3-labeled siRNA/polymer complexes, in solution, by dynamic light scattering. For the cationic MPC polymers, PMA37 and PMBA154M, the mean aggregate size was smaller in the presence of siRNA, compared to the absence of siRNA. However, PMB37 showed almost the same aggregate size, with or without siRNA. When siRNA was added to the cationic MPC polymer solution, the cationic unit of the MPC polymer and the siRNA interacted to form an aggregate complex. In addition, for all three types of MPC polymer, a smaller aggregation size was observed in those having a large proportion of the BMA unit, both in the presence and absence of siRNA.

The hydrophobic BMA units might interact with each other in aqueous solution, so that the polymers associated to form a much denser aggregate or complex. From these results, it was concluded that MPC polymers with a cationic unit were complexed with siRNA in the solution. It was also found that the MPC polymer with a hydrophobic unit forms an aggregate or complex with a smaller aggregate size. The ζ-potential of siRNA/MPC polymer complex reflects polymer chemical structure, that is, the siRNA/PMB37 complex took the value almost zero mV. It was the same as the PMB37 without siRNA as reported previously [32]. The PMA37 and PMBA154M had positive values even when they complexed with anionic siRNA.

### 3.4. Transportation of siRNA/MPC Polymer Complex into Cells

**Figure 5:**
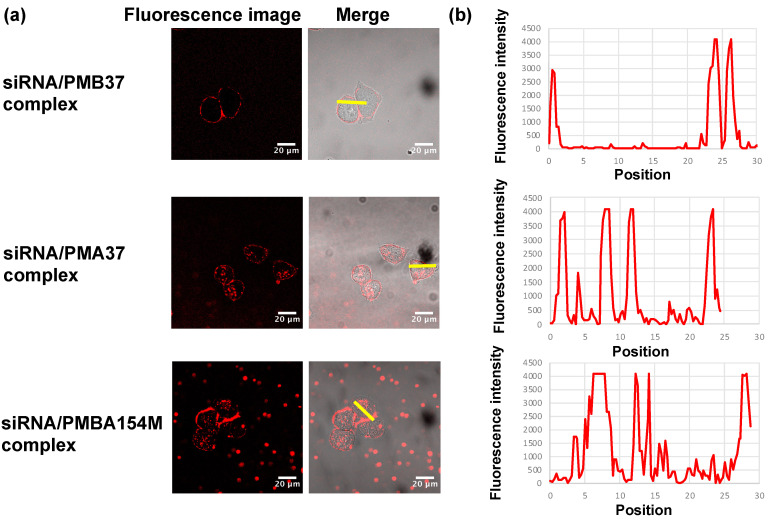
(**a**) Laser confocal microscopic images of HeLa-Luc cells internalized with various siRNA/MPC polymer complexes. Cells incubated in the medium containing the Cy3-labeled siRNA/MPC polymer complex at 37 °C for 20 min. (**b**) Line profile of fluorescence intensity of cells incorporated with Cy3-labeled siRNA/MPC polymer complex.

Observations of Cy3-labeled siRNA after applied to the cells with a confocal microscope are shown in Figure 5a. Fluorescence intensity profiles were evaluated in a cross-section of cells and the results are shown in Figure 5b. When only siRNA was added to the medium, intracellular fluorescence of siRNA was not observed (Figure S2), demonstrating that siRNA alone is not incorporated into cells. However, in the presence of some of the MPC polymers, fluorescence derived from siRNA was observed intracellularly. This observation indicated that the siRNA was taken up into the cells, in the presence of MPC polymer. It was concluded that, in these conditions, the MPC polymer has the ability to transport siRNA into cells.

We evaluated the effect of the chemical structure of the polymers on their siRNA transportation behavior. When PMA37 and PMBA154M, containing an AEMA unit, were used, siRNA was incorporated into cells, but when PMB37 was used, without AEMA, siRNA was not incorporated. To clarify, it was possible to incorporate siRNA into cells by using a polymer with a cationic AEMA unit, capable of interacting with siRNA. The siRNA is a hydrophilic polyanion, carrying a negative charge in the molecule, which can interact with a substance having a cationic site. Polymers with cationic monomer units are thought to complex by interacting with siRNA (Figures S3 and S4, and Table 2). The siRNA/polymer complex would then interact with the cell membrane and be taken into the cell, due to the effect of the cationic and hydrophobic units in the polymer. Although PMB37 itself was taken up into the cell, siRNA could not be transferred into cells.

**Figure 6:**
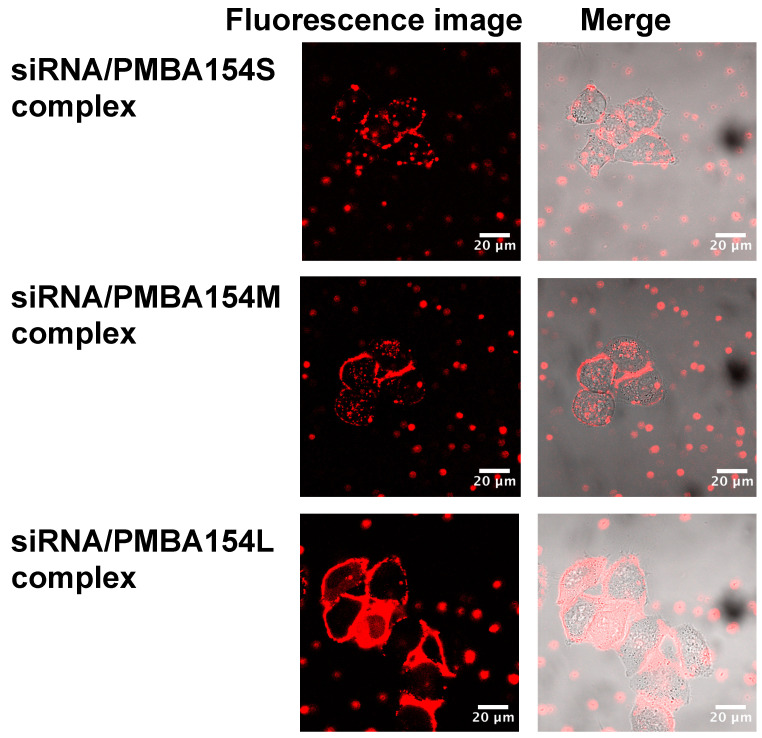
Effect of molecular weight of the MPC polymer on internalization of Cy3-labeled siRNA to HeLa-Luc cells. Cells were incubated at 37 °C.

As shown in Figure 6, the intracellular uptake of siRNA was observed when PMBA154S, PMBA154M, and PMBA154L, of different molecular weights, were used. In addition, the cationic homopolymers, PAEMA and PTMAEMA also showed the ability to carry siRNA into cells. From these findings, we suggested that a polymer containing 50% or more of a cationic unit in the molecule and having a Mw of about 10^5^–10^6^ has the ability to transport siRNA into cells.

To understand transportation pathway of the siRNA/MPC polymer complex through the cell membrane, uptake of Cy3-labeled siRNA in a 4 °C environment was observed using with a confocal microscope (Figure S5). The fluorescence derived from siRNA was observed around the cell membrane in the presence of PMBA154M or PMA37, but no fluorescence was observed inside the cells. Therefore, these MPC polymers did not transfer siRNA into cells at 4 °C. It is known that, when solute uptake into cells occurs by endocytosis, which uses metabolic energy, the solute uptake is not observed at 4 °C [20,47]. As these MPC polymers showed the ability to transfer siRNA into cells at 37 °C, we concluded that endocytosis was the dominant transportation pathway of the siRNA/MPC polymer complex.

### 3.5. Effects of siRNA/MPC Polymer Complex on Functionality of Cells

HeLa-Luc cells constantly express the luciferase gene [30]. Luciferase is an enzyme that catalyzes a chemical reaction in which the substrate d-luciferin emits light. In this reaction, d-luciferin first decomposes adenosine triphosphate (ATP) into adenosine monophosphate (AMP) and phosphate to produce the d-luciferin-AMP intermediate. Then, d-luciferase and the intermediate react with each other to generate excited state oxyluciferin, and light is emitted when returning to the ground state. When the amounts of d-luciferin and ATP are constant in this reaction, the luminescence intensity depends on the amount of luciferase. Therefore, it is possible to evaluate the state of expression of the luciferase gene by quantitatively analyzing the luminescence, in cells expressing the luciferase gene [48]. In this study, we used this reaction to evaluate the ability of MPC polymers to introduce siRNA into cells. When siRNA is introduced into HeLa-Luc cells, the activity of siRNA suppresses the expression of the luciferase gene.

**Figure 7:**
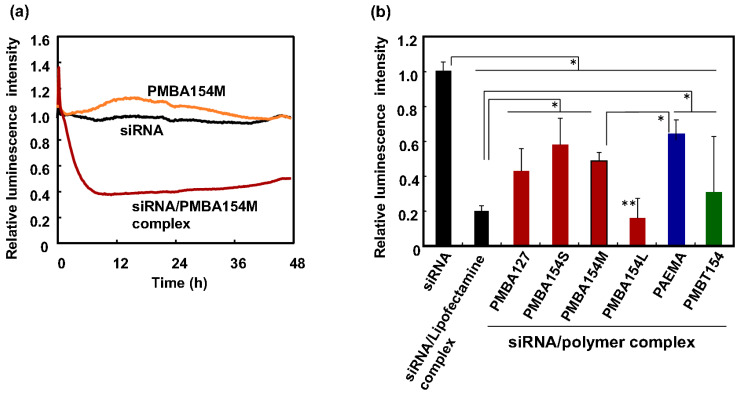
(**a**) Time course of relative luminescence intensity of HeLa-Luc cells by addition of polymer and GL3-labeled siRNA/polymer complex. (**b**) Relative luminescence intensity of HeLa-Luc cells by addition of various GL3-labeled siRNA/polymer complexes after 48-h incubation (*n* = 3, SD). * represents *p* < 0.01 (significant difference) and ** represents *p* > 0.01versus siRNA/Lipofectamine complex (not significant difference).

Next, we evaluated the level of gene suppression arising from transportation of GL3-siRNA into cells, using the polymers. Figure 7a shows a graph of time-dependent change in relative luminescence intensity by real-time measurement. The luminescence intensity was normalized using untreated cells, and the relative intensity was plotted on the vertical axis. When only siRNA or PMBA154M without siRNA was added, the intensity of luminescence did not decrease, and there was almost no difference from untreated cells. However, when a solution of the siRNA/PMBA154M complex was added, the intensity of luminescence decreased. This demonstrates that siRNA was transferred into the cells using with PMBA154M, to induce the RNA interference effect. The relative luminescence intensity initially decreased to about 40% by addition of siRNA/PMBA154M complex, and remained at about 50%, 48 h after the addition. From these results, we concluded that the siRNA/PMBA154M complex had a gene suppression efficiency of about 40–50%. In summary, PMBA154M, with its cationic and hydrophobic units, could be used to introduce siRNA into a cell and produce a gene suppression effect.

Figure 7b shows the gene suppression efficiency of GL3-siRNA into cells, by using various MPC polymers. The siRNA/Lipofectamine complex induced a remarkable decrease in relative luminescence. It was also found that the luminescence intensity decreased when siRNA/PAEMA complex was used.

The siRNA/PMBA154M complex showed a greater inhibition of luminescence than the siRNA/PAEMA complex. Since PMBA154M has a hydrophobic unit, it is considered to have high affinity with the lipid bilayer of cell membranes. As a result, siRNA was easily taken up into the cytoplasm, and the intracellular siRNA was sufficient to suppress gene expression. Therefore, we conclude that the siRNA/PMBA154M complex suppressed gene expression. Moreover, when the effect of the molecular weight of the PMBA154 series was evaluated, it was observed that the relative emission intensity tended to decrease, dependent on the molecular weight. The siRNA/PMBA154L complex showed a large decrease in relative emission intensity as the same as that observed in the siRNA/Lipofectamine complex. Although the reason for this phenomenon is not clear, we hypothesized that the PMBA154 series used in this study had a stronger interaction force per molecule, as the molecular weight increases, and we considered that PMBA154L might interact more stably with siRNA, to better induce endocytosis at the membrane surface.

Differences were observed in the gene suppression efficiency, even among the various MPC polymers that showed, by fluorescence observation, the ability to transport siRNA (see Figure 3). It was shown that differences in parameters such as chemical structure and composition of monomer units, and molecular weight of the polymer, had an effect on siRNA transportation efficiency and cytotoxicity. In particular, PMBA154L with its AEMA unit as a cationic group, and having a M_w_ of about 10^6^, was found to have extremely low cytotoxicity, while having a gene suppression efficiency comparable to that of Lipofectamine. The reduced cytotoxicity might be due to the MPC units in the polymer. An amphiphilic phospholipid polymer bearing a hydrophobic group usually has lower solubility in an aqueous solvent as the molecular weight increases. However, the PMBA154L was considered to have improved solubility in an aqueous medium, due to the high water-solubility of the MPC unit. The MPC unit contributes to the improvement of cytocompatibility and water-solubility of the polymer. In contrast, an increase in the proportion of MPC units in the polymer suppresses the interaction between the polymer and cell membrane and the complexation with biomolecules. The mole fraction of MPC units in the PMBA154 series was 10–20%, which improved the cell compatibility, maintained its water-solubility, even after complexation with siRNA, and improved the interaction between siRNA and the cell membrane.

## 4. Conclusions

We synthesized water-soluble and amphiphilic MPC polymers, with various chemical structures, by controlling the chemical structure and composition of the monomer unit, and the molecular weight of the polymer. The cationic MPC polymer interacted with biomolecules such as siRNA and the cell membrane, and could transport siRNA into cells. The introduction of hydrophobic BMA units (20–50 mol%) into the cationic MPC polymer, enhanced the gene suppression caused by the transportation of siRNA. Also, 10–20 mol% of MPC units in the cationic MPC polymer provided cytocompatibility, while maintaining the interaction with siRNA and the cell membrane. The gene suppression efficiency of the MPC polymer was the same as a conventional siRNA transporter with less cytotoxicity. These findings are expected to enable the development of more efficient transporters of biomolecules to cells in vitro.

## Figures and Tables

**Table 1:** Synthetic results of polymers used in this study.

Polymers	Composition (MPC/BMA/AEMA/TMAEMA)		Initiator	Solvent	[Monomer] (M)	[Initiator] (mM)	Time (h)	Molecular Weight		
	In Feed	In Polymer						Mw	Mn	Mw/Mn
PMB37	0.30/0.70/0/0	0.29/0.71/0/0	PB-ND	EtOH	1.6	55	4.0	1.0 × 10^4^	7.4 × 10^3^	1.4
PMA37	0.30/0/0.70/0	0.29/0/0.71/0	AIBN	MeOH/H_2_O (90/10)	1.0	5.0	3.0	7.5 × 10^5^	2.7 × 10^5^	2.6
PMBA154S	0.10/0.50.0.40/0	0.18/0.38/0.44/0	AIBN	MeOH/H_2_O (90/10)	0.5	12.5	20	1.4 × 10^5^	9.5 × 10^4^	1.4
PMBA154M	0.10/0.50.0.40/0	0.12/0.53/0.35/0	AIBN	MeOH/H_2_O (90/10)	1.0	5.0	20	3.6 × 10^5^	2.1 × 10^5^	1.7
PMBA154L	0.10/0.50.0.40/0	0.18/0.36/0.46/0	AIBN	MeOH/H_2_O (90/10)	2.0	2.0	2.0	1.5 × 10^6^	7.2 × 10^5^	2.2
PMBA127	0.10/0.20/0.70/0	0.06/0.21/0.73/0	AIBN	MeOH/H_2_O (90/10)	1.0	5.0	20	3.2 × 10^5^	2.3 × 10^5^	1.4
PMT37	0.30/0/0/0.70	0.37/0/0/0.63	AIBN	EtOH	1.0	5.0	3.0	3.8 × 10^5^	2.2 × 10^5^	1.7
PMBT154	0.10/0.50/0/0.40	0.12/0.55/0/0.33	AIBN	EtOH	1.0	5.0	6.0	4.1 × 10^5^	2.5 × 10^5^	1.6
PMBT127	0.10/0.20/0/0.70	0.15/0.19/0/0.66	AIBN	EtOH	1.0	5.0	6.0	4.6 × 10^5^	2.3 × 10^5^	2.0
PAEMA	0/0/1.0/0	0/0/1.0/0	AIBN	MeOH/H_2_O (90/10)	1.0	5.0	3.0	3.3 × 10^5^	2.3 × 10^5^	1.4
PTMAEMA	0/0/0/1.0	0/0/0/1.0	AIBN	EtOH	1.0	5.0	3.0	6.5 × 10^5^	3.5 × 10^5^	1.9
f-PMB37	0.30/0/70/0/0 0.1 ppm of FLMA	0.27/0.73/0/0 + FLMA	PB-ND	EtOH	1.0	55	18	1.1 × 10^4^	5.7 × 10^3^	2.0
f-PMA37	0.30/0/0.70/0 0.1 ppm of FLMA	0.28/0/0.72/0 + FLMA	AIBN	MeOH/H_2_O (90/10)	1.0	5.0	3.0	4.4 × 10^5^	2.8 × 10^5^	1.6
f-PMA154	0.10/0.50/0.40/0/ 1.0 ppm of FLMA	0.18/0.36/0.46/0 + FLMA	AIBN	MeOH/H_2_O (90/10)	1.0	5.0	20	3.2 × 10^5^	2.0 × 10^5^	1.6

**Table 2:** Size and ζ-potential of siRNA/MPC polymer complex.

Polymers	Volume-Means Aggregate Size (nm)(PDI)		ζ-Potential (mV)
	Polymer	SiRNA/Polymer Complex	SiRNA/Polymer Complex
PMB37	4.5 ± 0.2(0.31)	4.2 ± 0.2(0.26)	−0.5 ± 0.1
PMA37	520 ± 52(0.74)	93 ± 26(0.55)	38 ± 5
PMBA154M	61 ± 21(0.47)	24 ± 6(0.31)	25 ± 6

PDI: polydispersity index.

## Supplementary Materials

The following are available online at https://www.mdpi.com/2073-4360/12/8/1762/s1, Figure S1: ^1^H-NMR spectra of representative polymers used in this study, Figure S2: Laser confocal microscopic images of HeLa-Luc cells after contact with Cy3-labeled siRNA at 37 °C, Figure S3: Laser confocal microscopic images of HeLa-Luc cells internalized various Cy3-labeled siRNA/MPC polymer complexes at 37 °C, Figure S4: Laser confocal microscopic images of HeLa-Luc cells internalized of Cy3-labeled siRNA/cationic polymer complexes at 37 °C, Figure S5: Laser confocal microscopic images of HeLa-Luc cells internalized of various Cy3-labeled siRNA/MPC polymer complexes at 4 °C.
