# Supplementary material for: Water-Soluble and Cytocompatible Phospholipid Polymers for Molecular Complexation to Enhance Biomolecule Transportation to Cells In Vitro
Source: Polymers (Basel). 2020 Aug 6;12(8):1762. doi: 10.3390/polym12081762 (PMC7465638; doi:10.3390/polym12081762)
Supplement: Supplementary file 1 [file polymers-12-01762-s001.pdf]

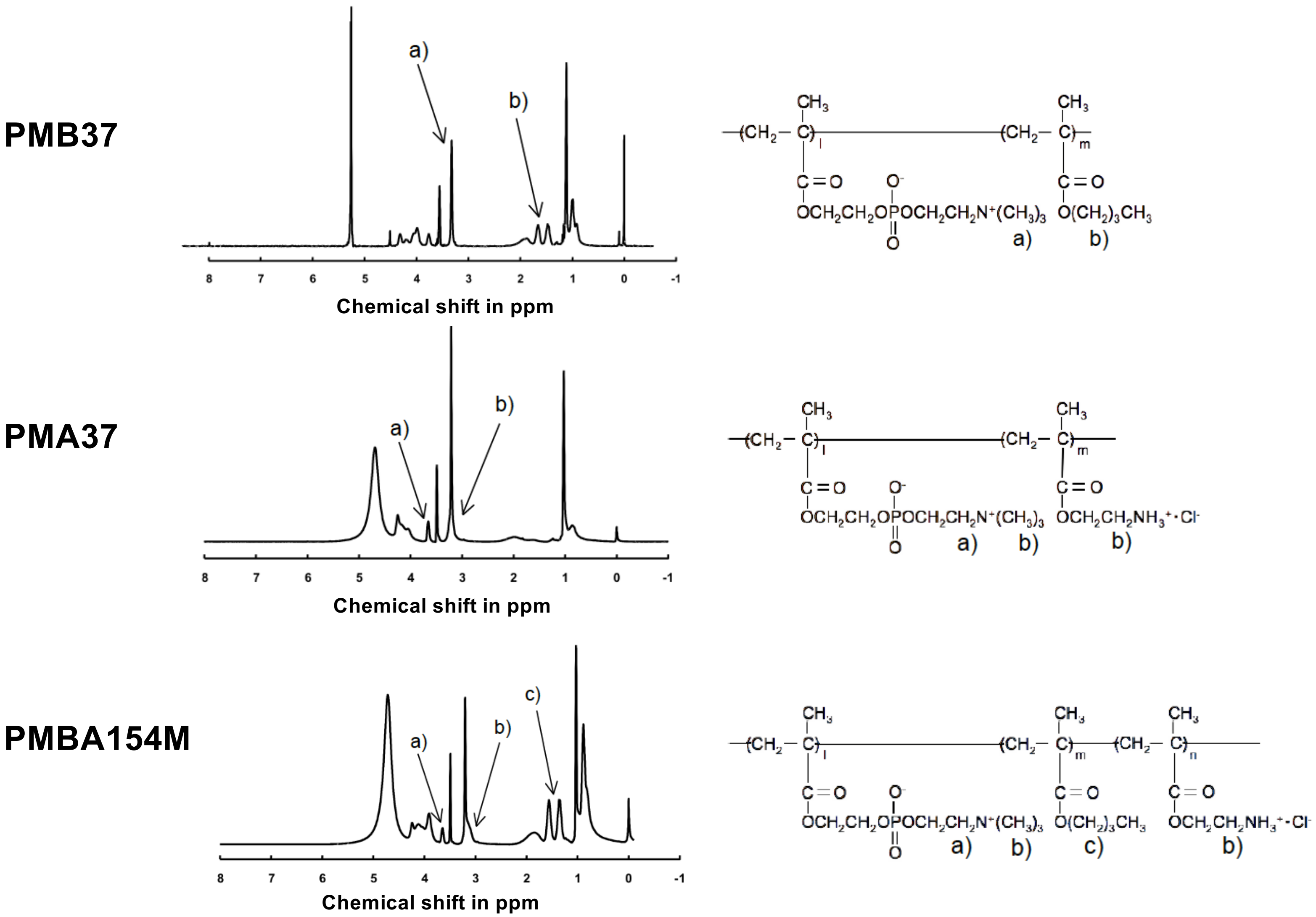

Figure S1. ^{1}H-NMR spectra of representative polymers used in this study.

siRNA only

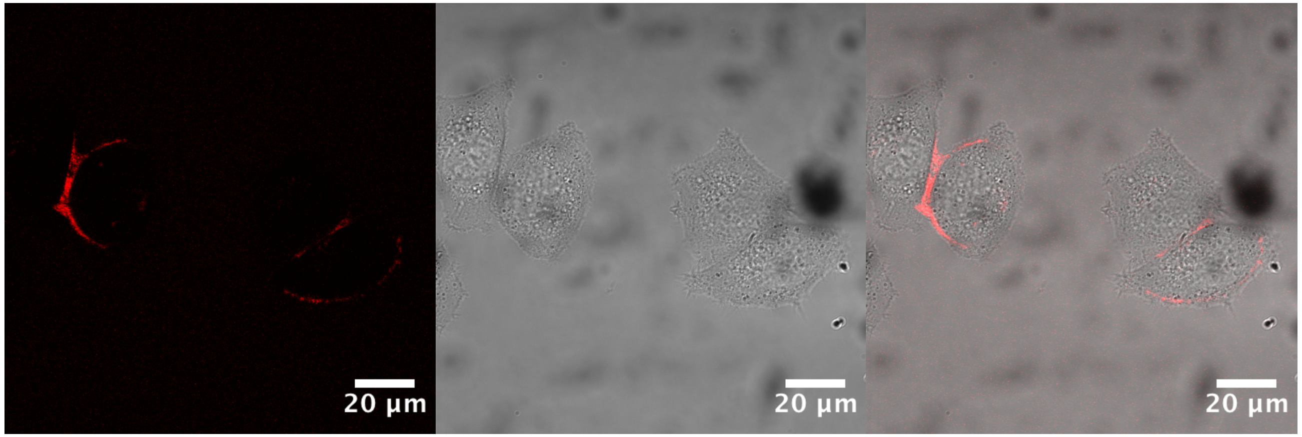

Figure S2. Laser confocal microscopic images of HeLa-Luc cells after contact with Cy3-labeled siRNA at 37 ˚C.

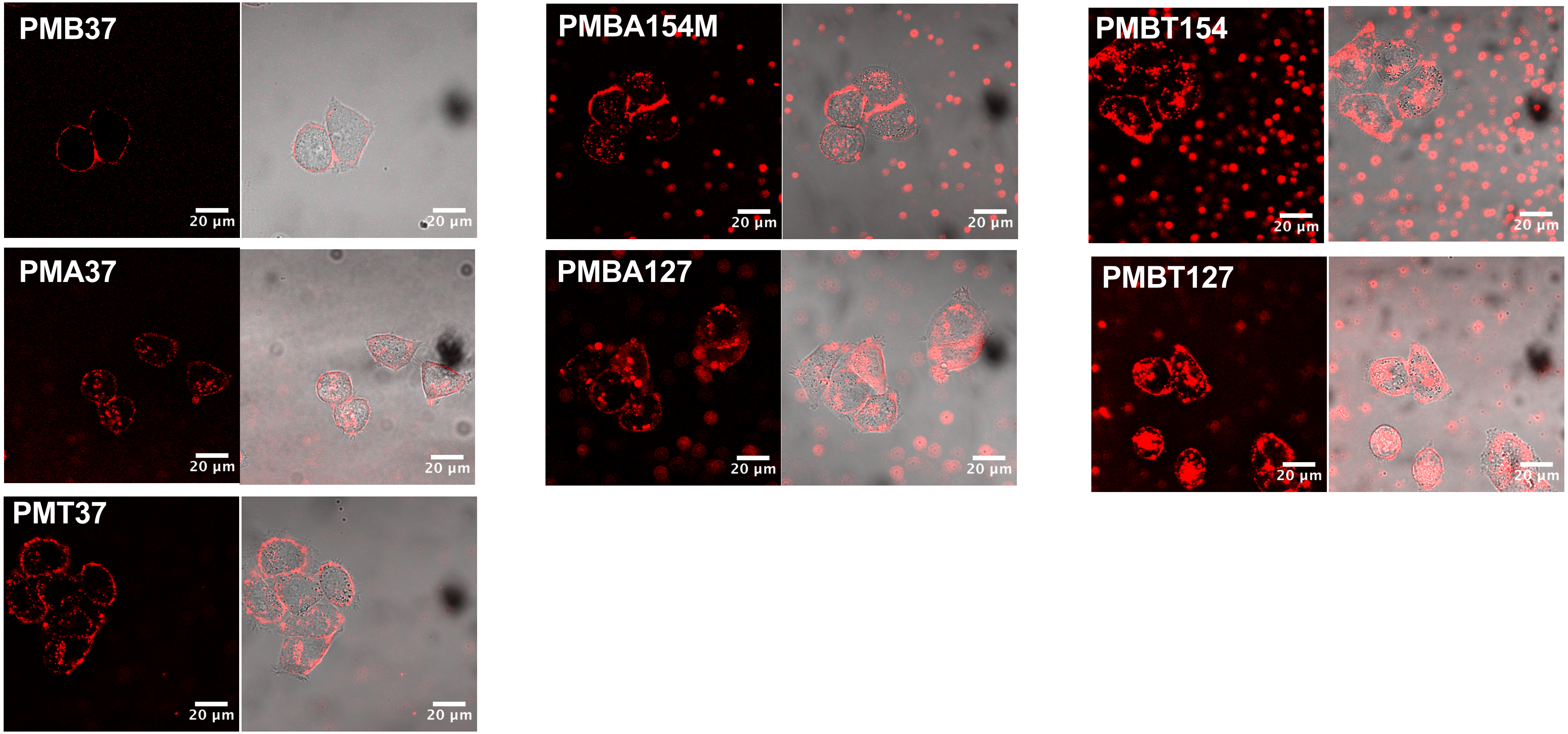

Figure S3. Laser confocal microscopic images of HeLa-Luc cells internalized with various Cy3-labeled siRNA/MPC polymer complexes at 37 ˚C.

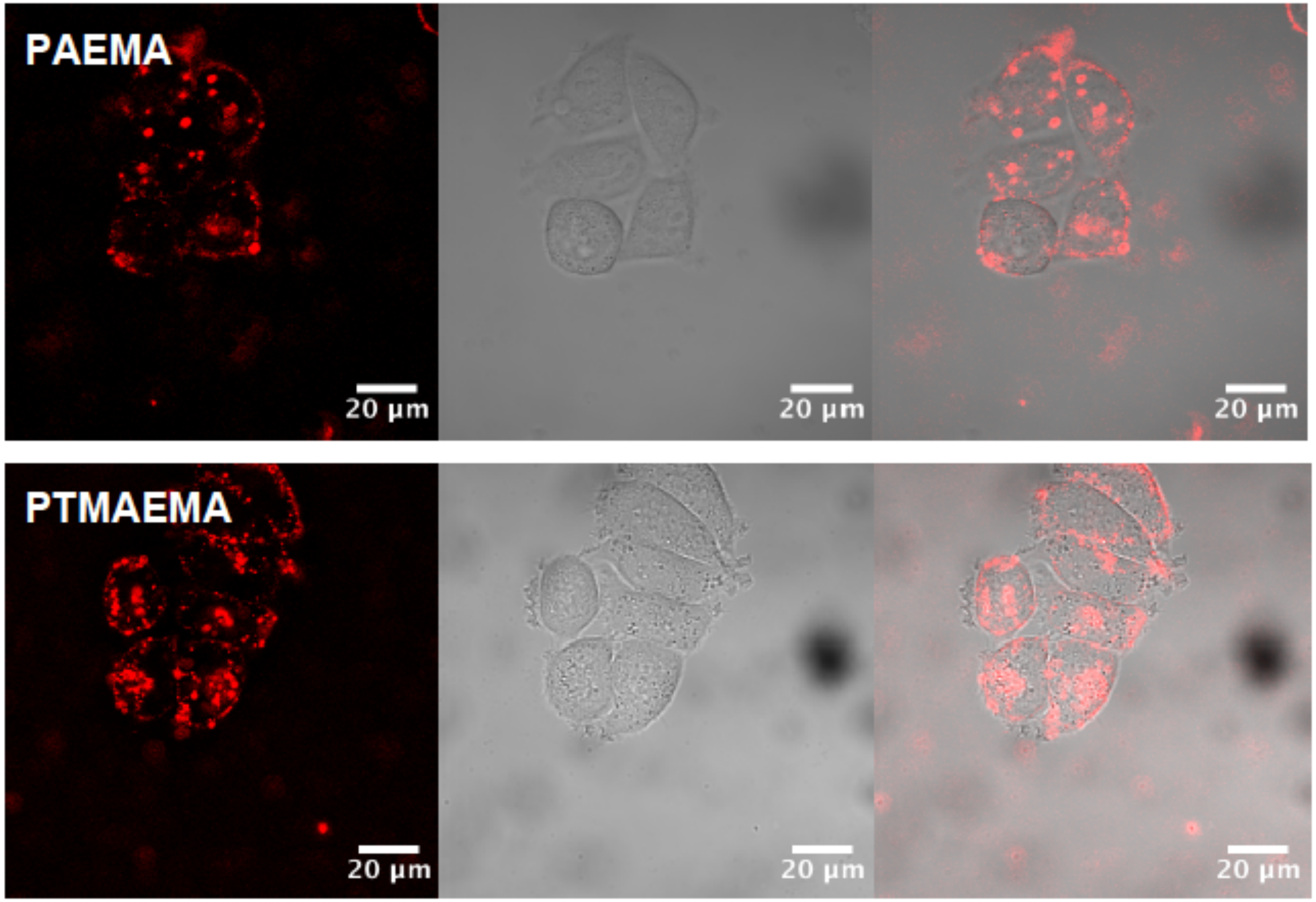

Figure S4. Laser confocal microscopic images of HeLa-Luc cells internalized with Cy3-labeled siRNA/cationic polymer complexes at 37 ˚C.

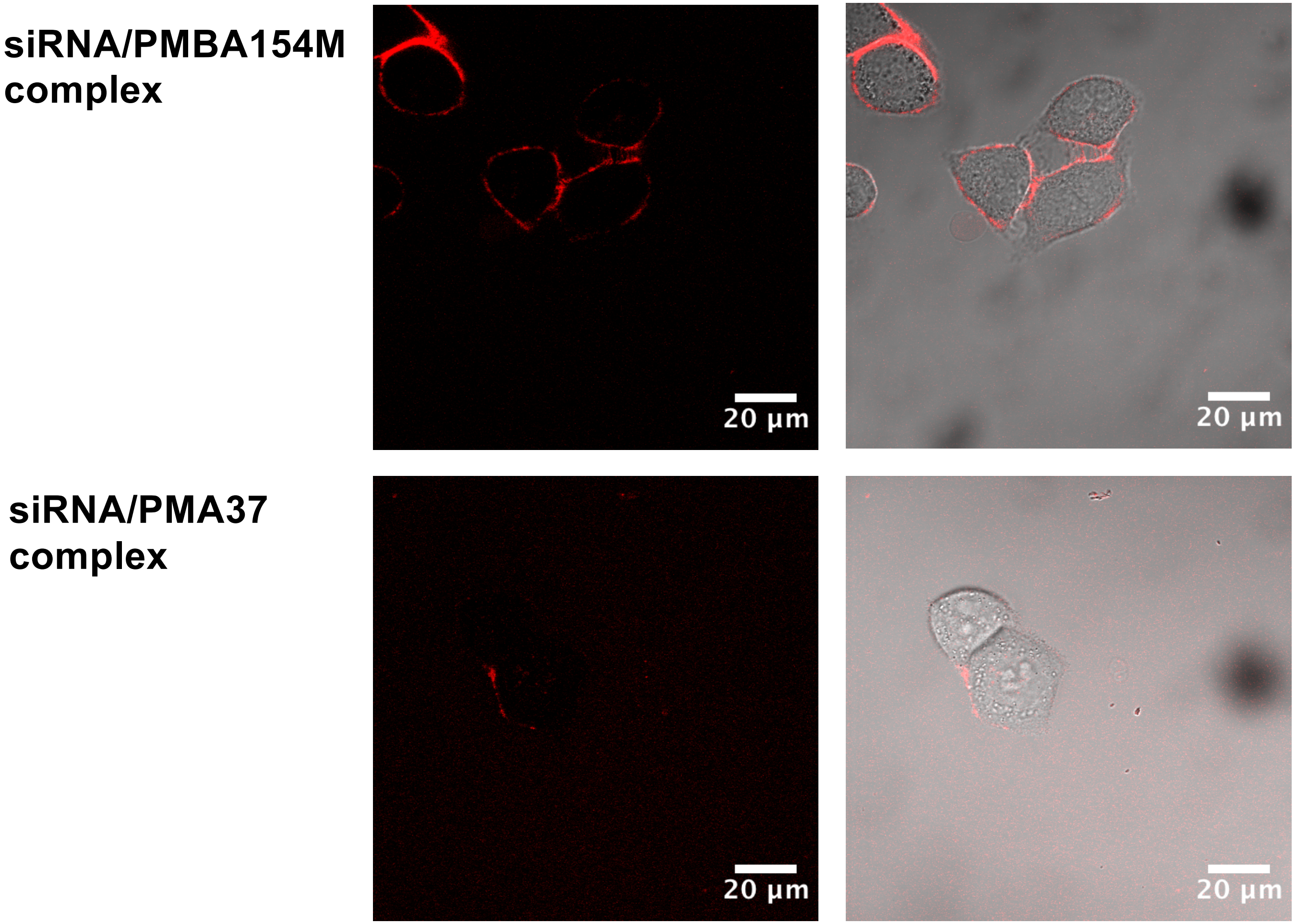

Figure S5. Laser confocal microscopic images of HeLa-Luc cells internalized with Cy3-labeled siRNA/cationic MPC polymer complexes at 4 °C.

Figure S5
